# High-Throughput Screening of the Thermoelastic Properties of Ultrahigh-Temperature Ceramics

**DOI:** 10.1021/acsami.1c08832

**Published:** 2021-06-16

**Authors:** Pinku Nath, Jose J. Plata, Julia Santana-Andreo, Ernesto J. Blancas, Antonio M. Márquez, Javier Fernández Sanz

**Affiliations:** † School of Chemical Engineering and Physical Science, Lovely Professional University, Phagwara 144411, India; ‡ Departamento de Química Física, Facultad de Química, Universidad de Sevilla, Seville 41012, Spain

**Keywords:** ultrahigh-temperature ceramics, UHTCs, thermoelasticity, high-throughput calculations, mechanical properties, extreme environments

## Abstract

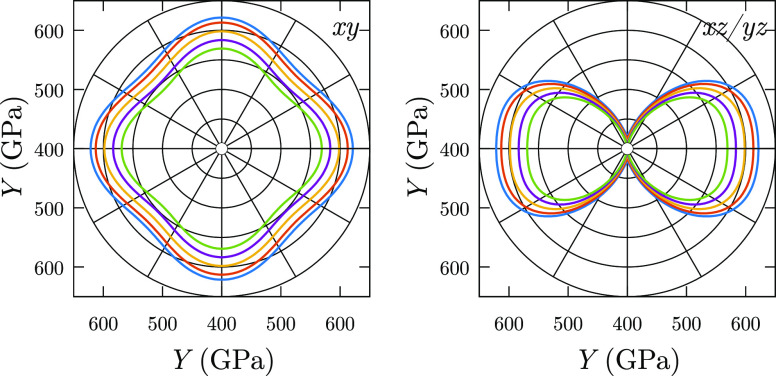

Ultrahigh-temperature ceramics (UHTCs) are a group of materials with high technological interest because of their applications in extreme environments. However, their characterization at high temperatures represents the main obstacle for their fast development. Obstacles are found from an experimental point of view, where only few laboratories around the world have the resources to test these materials under extreme conditions, and also from a theoretical point of view, where actual methods are expensive and difficult to apply to large sets of materials. Here, a new theoretical high-throughput framework for the prediction of the thermoelastic properties of materials is introduced. This approach can be systematically applied to any kind of crystalline material, drastically reducing the computational cost of previous methodologies up to 80% approximately. This new approach combines Taylor expansion and density functional theory calculations to predict the vibrational free energy of any arbitrary strained configuration, which represents the bottleneck in other methods. Using this framework, elastic constants for UHTCs have been calculated in a wide range of temperatures with excellent agreement with experimental values, when available. Using the elastic constants as the starting point, other mechanical properties such a bulk modulus, shear modulus, or Poisson ratio have been also explored, including upper and lower limits for polycrystalline materials. Finally, this work goes beyond the isotropic mechanical properties and represents one of the most comprehensive and exhaustive studies of some of the most important UHTCs, charting their anisotropy and thermal and thermodynamical properties.

## 1 Introduction

Ultrahigh temperature ceramics (UHTCs) are usually defined as compounds whose melting point surpasses 3000 °C.^1^ While UHTCs are not new materials for the scientific community and have been reported since late 1800s, their technological interest started to grow in the late 1960s. In the most recent decade, UHTCs have clearly emerged because of their potential use in extreme environments.^1^ Aerospace applications such as scramjet propulsion, hypersonic aerospace vehicles, and advanced rocket motors are the main reason why research on UHTCs has grown in recent years.^2^ For instance, thermal control, mechanical resistance, and corrosion are the main variables to consider when designing hypersonic vehicles, whose materials experience temperatures higher than 2000 °C and are exposed to highly reactive, dissociated gas species.^3^ UTHCs combine high hardness, stiffness, and melting temperature with very low reactivity because of their strong covalent bonds between carbon, nitrogen, or boron with transition metals such as Hf, Zr, Nb, Ti, or Ta.^1^

UHTC-based materials have been rapidly developed during the last 25 years, but there are still many challenges to be tackled in order to spur the rational design, synthesis, and deployment of these materials. The main issue that hampers the swift development of these materials is their experimental characterization and testing at extreme environments. Most well-known properties of these materials are obtained under ambient conditions, and there are few laboratories around the world with the resources to test these materials under extreme conditions.^4^ Thus far, computational approaches have not presented solutions to these experimental barriers. Most of the theoretical works related to UHTCs are focused on 0 K properties,^5^ and there are few reports in which temperature-dependent mechanical properties of UHTCs are explored.^6,7^ This has been due to (i) the scarcity of commercial algorithms to predict these temperature-dependent properties and (ii) the high computational cost of these calculations.

During the last decade, the development of high-throughput frameworks for the prediction of mechanical properties has changed the pace at which materials are discovered and characterized. For instance, the Automatic GIBBS Library has been able to predict mechanical and thermodynamic properties such as bulk modulus of thousands of materials at a very low computational cost.^8^ While this method quantitatively describes the mechanical properties of isotropic materials with few components accurately, their results are mainly useful from a qualitative point of view. There are methods, such as the quasi-harmonic approximation (QHA),^9,10^ that provide a relatively affordable computational approach to obtain temperature-dependent mechanical properties, obtaining good quantitative agreement with experimental results. However, QHA is most frequently used with isotropic volume deformations of the crystal, so the properties obtained through this method can be considered as average mechanical features of the system. This is the reason why elastic constants need to be computed in order to fairly capture the anisotropy of the material and obtain a more complete description of the temperature-dependent mechanical response of the system.^11^ Thus, elastic constants represent the starting point that gives access to other mechanical properties. There are also high-throughput frameworks that predict elastic constants at 0 K such as ElaStic^11^ or AEL,^12^ however, including temperature effects increases the complexity of the theoretical framework and also the computational cost. Different approaches have been proposed to compute temperature-dependent elastic constants using QHA as a formal framework.^13−16^ VLab represents a good example of a robust framework that can include temperature effects on the prediction of elastic constants.^17,18^ Nevertheless, their computational costs prevent them from being used systematically or routinely. Other methods have been developed in order to reduce the cost of using the QHA to compute temperature-dependent elastic constants.^19−21^ For instance, the quasi-static approximation (QSA) reduces the number of calculations, assuming that the temperature dependence of the elastic constant is primarily due to thermal expansion.^19,20^ Still, QSA tends to underestimate thermal effects and increase anisotropy, which is detrimental to its use, especially at high temperatures.^22^ Similarly, PS-QHA represents another inexpensive methodology to predict the elastic constants through a self-consistent minimization of the total pressure.^21^ Nevertheless, this approach, based on SC-QHA,^20^ overestimates the thermodynamic properties, particularly at high temperatures.^23^ Developing a high-throughput framework that predicts temperature-dependent elastic constants and combines accuracy and robustness while also reducing computational costs remains a challenge.

In this work, we have extended the three-phonon approach, QHA3P method,^23^ to calculate temperature-dependent elastic constants combining Taylor expansion with QHA. Following this strategy, Taylor expansion reduces the computational cost, while mechanical and thermodynamical properties at a particular temperature are determined by minimizing free energy. The elastic constants of UHTCs at finite temperatures are predicted to chart their mechanical properties, paying special attention to the high temperature range, in order to simulate their behavior under extreme conditions, which determines their potential application in industry. To do so, a new high-throughput framework has been designed that not only automatizes the process but also includes a new approach that reduces the computational cost compared with previous methodologies up to 80%, without losing accuracy.

## 2 Methodology

### 2.1 Elastic Constants

Traditionally, elastic properties can be described within the Lagrangian theory of elasticity in which a solid is considered as a homogeneous and anisotropic elastic medium. Within a linear regime and using the Voigt notation, the stress, **σ** = (σ_1_, σ_3_, σ_3_, σ_4_, σ_5_, σ_6_), and strain, **ϵ** = (ϵ_1_, ϵ_2_, ϵ_2_, ϵ_2_, ϵ_2_, ϵ_6_), relation can be expressed as^22,24^
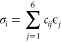
1where *c*_*ij*_ are elastic stiffness constants of a crystal represented in a 6 × 6 matrix where *c*_*ij*_ = *c*_*ji*_. Considering this constraint, the total number of independent elastic components is 21 instead of 36. Alternatively, it is possible to define the total energy of a crystal in terms of a power series of the strain^11^ as
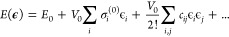
2where *E*_0_ and *V*_0_ are the density functional theory energy and volume of the reference structure, respectively. If the optimized (ground state) structure is chosen as the reference, σ_*i*_^(0)^ = 0 because the equilibrium structure is stress-free.

Two alternative expressions can be derived for the elastic constants according to eqs 1 and 2
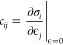
3and
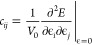
4

Methods based on eq 3 to calculate *c*_*ij*_ are defined as “stress approach”, while methods based on eq 4 are classified as “energy approach”. Although both are based on the creation of strained structures, there are important differences between them. The stress–strain approach is the most used method and a lower number of strained structures are needed. However, time-consuming calculations are required to obtain the same accuracy as with the results obtained with the energy-strain method using a less demanding setup. That is why the energy-strain method is preferred to reduce the sensitivity of the results with respect to the calculation setup.

To compute temperature-dependent elastic constants, a similar approach as shown in eq 4 can be used. Still, it requires the calculation of the free energy and the temperature-dependent equilibrium volume. The QHA is one of the methods that gives access to compute free energy and equilibrium properties.

### 2.2 Combining QHA and Elastic Constants

**Figure 1:**
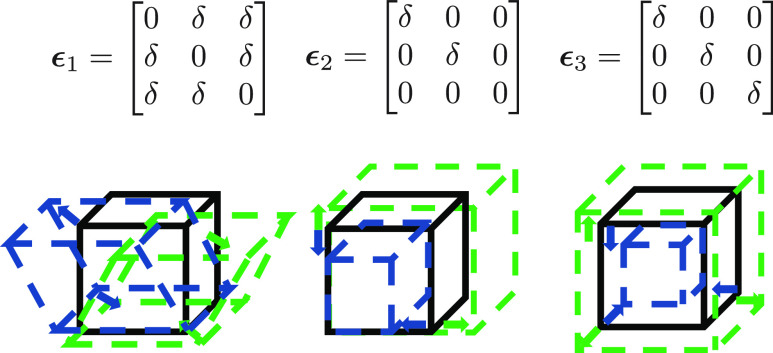
Strain tensors for a cubic system and their effect on the lattice vector of the crystal. Distorted cells represented by dashed green lines are obtained when δ > 0, and distorted cells represented by dashed blue lines are obtained when δ < 0.

In QHA, total free energy of a system, *F*_tot_, is described as a sum of three terms (eq 5): (i) vibration-less total energy at 0 K, *E*_0_, (ii) the vibrational free energy, *F*_vib_, and (iii) free energy due to thermal electronic excitation, *F*_elec_. Strain-dependent *F*_tot_ can be described as^22,25^
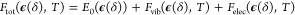
5where *F*_tot_, *F*_vib_, and *F*_elec_ are functions of the applied strain, **ϵ**(δ), and temperature, *T*. The applied strain contains structural information and it is a function of the amplitude of distortion, δ. Examples of strain tensors for a cubic system are shown in Figure 1. The first term of the equation can be computed with different *ab initio* packages. The second term is obtained integrating over the phonon density of states (DOS). The last term of the equation is calculated integrating over the electronic DOS.

The calculation of *F*_vib_ at a given **ϵ** is performed using the harmonic approximation, where *F*_vib_ includes anharmonic effects in the form of strain-dependent phonon frequencies^10,23,25^
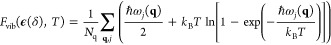
6where *ℏ* and *k*_B_ are the reduced Planck and Boltzmann constants, respectively, and ω_*j*_(**q**) is the strain-dependent phonon frequency for the wave vector, **q**, and phonon branch index *j*. *N*_q_ is the total number of wave vectors.

For metals and narrow band gap systems, the contribution of *F*_elec_(**ϵ**(δ),*T*) to *F*_tot_ could be important and includes temperature-dependent contribution of the electrons to the internal energy, *U*_elec_(**ϵ**(δ),*T*), and the electronic entropy, *S*_elec_(**ϵ**(δ),*T*)^23,25^
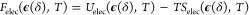
7

Both terms can be calculated as
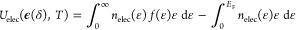
8and
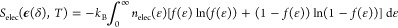
9where *n*_elec_(ε) is the density of states at energy ε, *f*(ε) is the Fermi–Dirac distribution, and *E*_F_ is the Fermi energy.

The temperature-dependent isothermal elastic constants, *c*_*ij*_^T^(*T*), can be obtained by minimizing temperature-dependent free energy, *F*(**ϵ**(δ), *T*), with respect to strain using a similar methodology, as shown in eq 4.

### 2.3 Accelerated QHA (QHA3P) and Strain

The computation of *F*_vib_ is the most time-consuming step when using QHA. Very recently, QHA3P has been developed as an inexpensive alternative to QHA.^23^ It has been already demonstrated that using QHA3P, isotropic thermodynamic properties are calculated, reducing the cost to one-third of the computational resources needed by QHA. In this approach, the free energy is expressed as a function of isotropic distortions such as volume, as well as temperature. Since the distortions are small enough, phonon frequencies around the relaxed configuration can be described using a Taylor series. Taylor expansion involves the computation of Taylor coefficients using three full phonon calculations, which are computed for two different distorted structures and one at the minimum of the potential energy surface (relaxed structure). Once these coefficients are computed for all wave vectors at the Brillouin zone, these are further used to estimate phonon frequencies for any arbitrary distortions. Therefore, QHA3P does not require computation of phonons for more than two distortions unlike QHA.

In this work, a similar approach has been used to reduce the computational cost of calculating strain-dependent *F*_vib_ (second term in eq 5). Here, *F*_vib_ is a function of structure-dependent parameter **ϵ** instead of isotropic distortions, and **ϵ** is a function of δ. For small values of δ (small distortions), Taylor expansion can be used around **ϵ**(0). Similarly to isotropic distortions, Taylor coefficients are calculated for all wave vectors at the Brillouin zone at each **ϵ** using only three phonon calculations (eq 10). Two of the phonon calculations are computed at positive and negative δ values and the third phonon calculation is computed at the unstrained structure (relaxed structure) with **ϵ**(0). These Taylor coefficients are again further used to compute phonon frequencies for arbitrary δ values, thus reducing the computational cost.
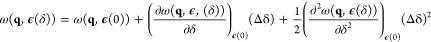
10

If *m* represents the number of different strain tensors **ϵ**_*i*_ and *n* is the number of distorted structures for a specific strain tensor, the number of phonon calculations using QHA will be *m* × (*n* – 1) + 1. However, for QHA3P, this number will be (2 × *m* + 1). For a cubic crystal, *m* = 3, so the number of phonon calculations is 37 and 7 for QHA and QHA3P, respectively (considering *n* = 13). This is an important reduction in computational time in comparison with QHA which is even larger for space groups with lower symmetry.

### 2.4 Workflow

**Figure 2:**
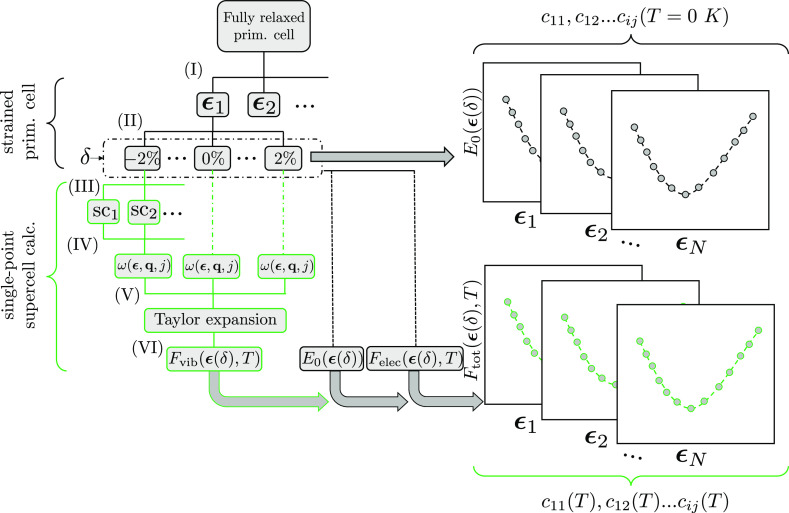
Workflow for calculating temperature-dependent elastic constants. (I) Identification of crystal symmetry and strain tensors. (II) Generations of distorted geometries and calculation of *E*_0_ and *F*_elec_. (III) Creation of supercells for phonon calculations. (IV) Frequency calculation for selected distorted structures. (V) QHA3P. (VI) Calculation of *F*_vib_ and *F*_total_ in order to extract elastic constants.

A high-throughput framework has been developed to automate the calculation of the temperature-dependent mechanical properties of UHTCs (Figure 2). This new approach combines the energy-strain method that is used in many high-throughput frameworks that computes 0 K elastic constants, with the QHA3P method that includes the vibrational energy contributions at any temperature but drastically reducing the computational cost. First, the primitive cell is fully optimized to characterize the minimum of the potential surface energy at 0 K. The energy-strain method is used to calculate the elastic constants of each material. Space groups for each material are calculated using the spglib library, determining the distortion matrices or strain tensors that will be applied to create the distorted cells. The set of distortion matrices has been chosen following the Zhang and Zhang approach.^24^ For instance, the distortions for a cubic system are depicted in Figure 1, where each strain tensor is a function of δ, which is a scalar that represents the magnitude of the strain. *n* strained cells are generated for each strain tensor, (*n* – 1)/2 of them with a δ < 0 and (*n* – 1)/2 of them with δ > 0. Once the distorted structures are created, *E*_0_, *F*_vib_, and *F*_elec_ energies need to be calculated for all distorted structures. The calculation of the frequencies used in eq 6 is the bottleneck of the process. The QHA3P method was applied in order to reduce the number of phonon calculations required to finally compute *F*_tot_(**ϵ**). Thus, only three phonon calculations, including two distortions and the fully optimized geometry (δ = 0), are required for a given **ϵ**_*i*_. QHA3P uses the phonon spectra and frequencies obtained from these three structures to estimate other frequencies for any arbitrary δ values for that particular **ϵ**_*i*_ using a Taylor expansion. This approach reduces the computational cost between 80 and 83% with respect to traditional approaches, depending on the crystal symmetry. Finally, *F*_tot_(**ϵ**, *T*) is fitted with a cubic polynomial to extract elastic constants.^22^ In order to facilitate the comparison and use of the results obtained in this work, all the properties calculated with this framework will be available on NewMaterialsLab website (https://www.newmaterialslab.com).

### 2.5 Isothermal and Adiabatic Elastic Constants

As mentioned previously, isothermal elastic stiffness constants, *c*_*ij*_^T^(*T*), can be calculated at finite temperatures by substituting internal energy, *E*, by free energy, *F*, in eq 4. However, from an experimental point of view, elastic constants are generally obtained in adiabatic rather than isothermal conditions, using techniques such as ultrasonic measurements or Brillouin scattering experiments. Adiabatic elastic constants, *c*_*ij*_^S^(*T*), are always equal to or larger than *c*_*ij*_^T^(*T*). In order to compare with experiments, *c*_*ij*_^T^(*T*) are converted into *c*_*ij*_^S^(*T*) following the relation reported by Davies^13^
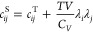
11where
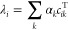
12with α_*i*_ being the linear thermal expansion coefficient in direction *i*, *C*_*V*_ being the specific heat, and ρ being the density. For cubic systems^22^
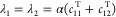
13and λ_4_ = 0, so *c*_44_^S^ = *c*_44_^T^. For hexagonal systems
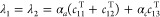
14and
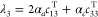
15where α_*a*_ and α_*c*_ are the linear thermal expansion coefficients in directions *a* and *c*.

### 2.6 Computational Details

#### 2.6.1 Geometry Optimization

All 0 K ground-state structures were fully relaxed (atoms and lattice) using VASP package.^26^ Energies were obtained combining the projector-augmented wave potentials^27^ with the exchange–correlation functional proposed by Perdew–Burke–Ernzerhof.^28^ All calculations use a high-energy cutoff, 700 eV for borides and 550 eV for carbides and nitrides. Reciprocal space was explored using a dense **k**-point mesh of 12,000 **k**-points per reciprocal atom, approximately. The wave function was converged self-consistently until the energy difference between two consecutive electronic steps was smaller than 10^–9^ eV. Partial occupancies for each orbital were determined using the Methfessel–Paxton method of order one. Geometry optimizations were performed using three-atom primitive cells for borides and eight-atom conventional cells for carbides and nitrides. Structures were considered fully relaxed when forces over all atoms were smaller than 10^–8^ eV/Å. An additional support grid for the evaluation of the augmentation charges was included to reduce the noise in the forces. In order to promote reproducibility, these structures will be available in 2021 in the ioChem-BD platform.

#### 2.6.2 Distorted Cells

Elastic constant prediction requires three and five distortion modes for cubic and hexagonal systems, respectively. These distortion modes are generated using the procedure shown elsewhere.^24^ The values assigned to δ go from −2 to +2% of each lattice vector, obtaining the 13 strained cells for each **ϵ**_*i*_. These generated structures are relaxed without changing the cell volume. After the relaxation, single-point calculations were performed for each strained cell in order to calculate their DOS.

#### 2.6.3 Phonon Calculations

Different packages can be used to predict the vibrational spectra of solids, such as APL-AAPL^29^ or Phonopy.^30^ In this case, phonon calculations were performed combining Phonopy and VASP to obtain second-order interatomic force constants *via* the finite displacement approach. For each **ϵ**_*i*_, two phonon calculations are performed at ±2% distortions including one at the equilibrium structure. Forces were extracted from 4 × 4 × 4 supercells for borides (192 atoms) and 3 × 3 × 3 supercells for nitrides and carbides (216 atoms). The magnitude of the displacement to obtain the force constants was 0.01 Å. The same SCF convergence criteria followed in the optimizations were used for these calculations. Frequencies and other related phonon properties such as *F*_vib_ were calculated using a 31 × 31 × 31 **q**-point mesh that ensures their convergence.

## 3 Results and Discussion

### 3.1 Phonon Dispersion Curves

Phonon dispersion curves are an essential part to compute the vibrational contribution to the free energy and, simultaneously, give information about the stability of these materials. The absence of imaginary frequencies confirms the dynamic stability of UHTCs at 0 K (Figure S1). Moreover, our results are in good agreement with previous theoretical predictions and, most importantly, experimental data (Figure S1). Only small deviations were found for the borides, which can be attributed to the measurement of the phonon dispersion curve at finite temperatures and specific surfaces.^31^

### 3.2 Elastic Constants

**Figure 3:**
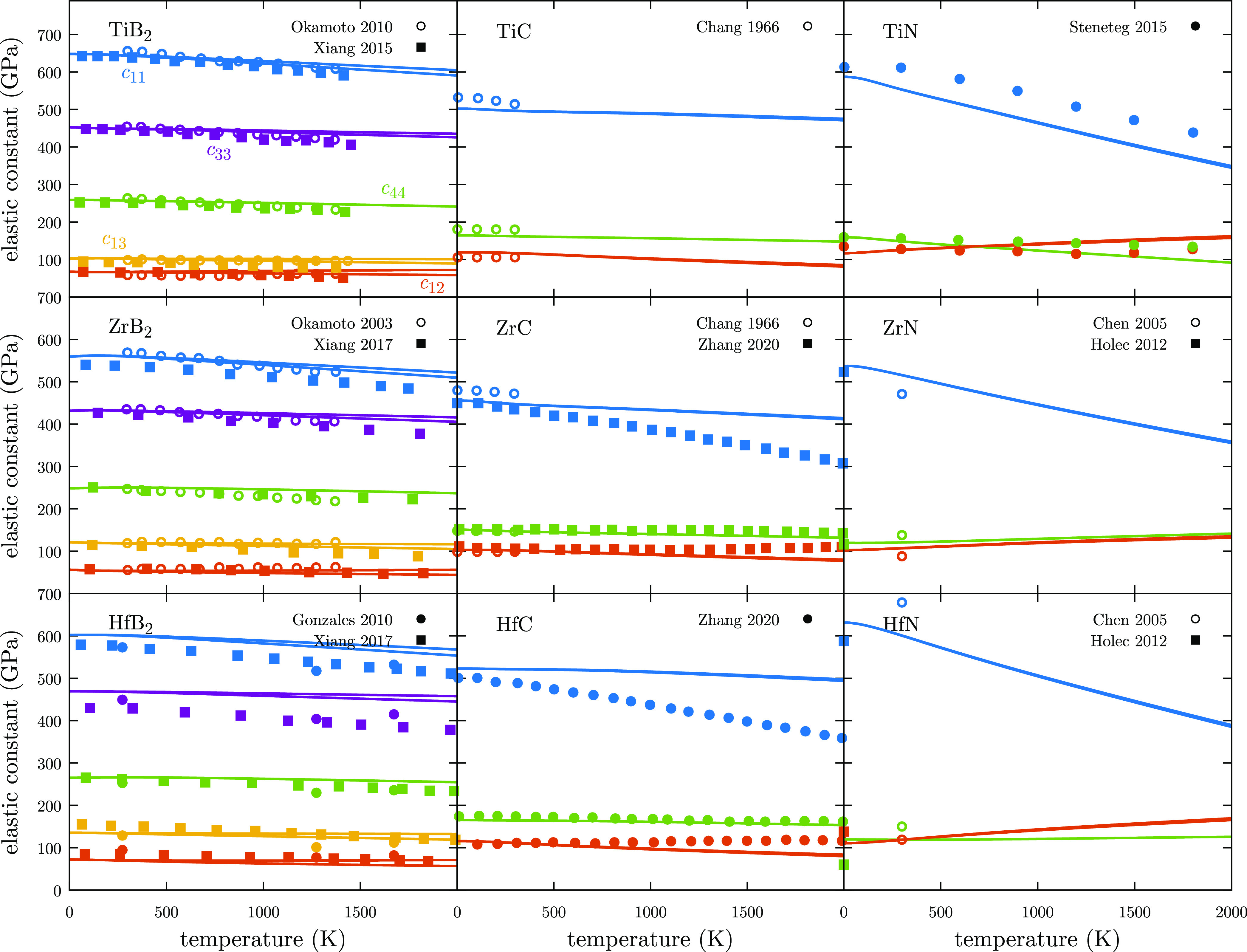
Isothermal (solid lines) and isentropic (dashed lines) elastic constants for UHTCs. Open points represent experimental measurements while filled points represent calculated values. Colors: *c*_11_ = blue; *c*_12_ = orange; *c*_13_ = yellow; *c*_33_ = purple; and *c*_44_ = green.

In this section, calculated isothermal and isentropic elastic constants are compared with previous experimental values and simulations that are available (Figure 3). To the best of our knowledge, TiB_2_ and ZrB_2_ are the only two UHTCs whose elastic constants have been experimentally well-characterized in a wide range of temperatures.^32,33^ The values obtained with the new high-throughput framework are in excellent agreement with the experiments and other calculated values obtained with more computationally demanding approaches.^7,34,91^ Experimental results, but in a shorter range of temperatures, were also found for TiC and ZrC,^35^ with relative errors always below 5%. Only room-temperature values have been reported for ZrN and HfN,^36^ so it is difficult to analyze any trends. That is why, we have also included calculated 0 K elastic constants for these two systems.^37^ In both materials, calculated and experimental available results are aligned with our results and only *c*_11_ at 300 K presents a small deviation. Only calculated values have been found for TiN^38^ and HfC.^39^ Molecular dynamics performed by Steneteg *et al.* seem to predict similar trends and values than the HT framework for TiN. For HfC, Zhang and McMahon obtained very similar values for *c*_12_ and *c*_44_ while *c*_11_ seems to decrease faster than in our results. However, they also obtained a very soft behavior of *c*_11_ for ZrC,^39^ while our methodology seems to follow the experimental measurements better. The fast reduction of *c*_11_ with temperature is more noticeable for nitrides. This trend is related to the changes in the volume when longitudinal strains are applied, which is strongly connected to temperature. Other elastic constants such as *c*_12_ and *c*_44_ are related to deformation resistance to strain modes that are not connected to big changes in the volume, so they are less affected by temperature. In addition to the comparison with previous reported values, elastic constants have also been calculated using the traditional QHA approach for HfC (Figure S2). There are not significant differences between the results obtained with QHA3P and traditional QHA in the whole range of temperature, but the new approach is approximately 80% less expensive. This is one of the characteristics that makes this framework so powerful. While other methods such as QSA also reduce the computational cost to compute the elastic constants, they lose accuracy, particularly at high temperatures.^39^

### 3.3 Mechanical Stability

Elastic constants describe the response of the crystal to external forces, so they play an important role determining their mechanical stability. Mechanical stability has been extensively explored by different theoretical and computational works. Here, Born stability criteria^40^ will be adopted to elucidate the mechanical stability of these materials in a wide range of temperatures. For a cubic crystal, the mechanical stability criteria under isotropic pressure are
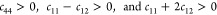
16

For hexagonal systems, the stability criteria are
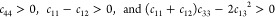
17

All the materials explored in this work fulfill the Born stability criteria in the studied range of temperatures (Figure S3).

### 3.4 Isotropic Mechanical Properties

Elastic constants are also the essential ingredient to compute some key isotropic mechanical properties such as bulk modulus, *B*, shear modulus, *G*, Young’s modulus *Y*, Poisson ratio σ, and hardness *H*_V_.

#### 3.4.1 Bulk and Shear Modulus

Different definitions have been proposed to calculate the bulk modulus of an aggregate of crystals. Voigt’s definition is based on the averaging of the relation expressing the stress in a single crystal over all possible orientations
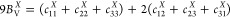
18where *X* = (S, T) in order to differentiate between adiabatic and isothermal values, respectively. While Voigt’s definition assumes that the strain is uniform through the aggregate, the Reuss approach considers that the stress is uniform
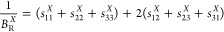
19where **s**^*X*^ = (**c**^*X*^)^−1^ is the compliance tensor, it has been proven that Voigt moduli are always larger that Reuss moduli with true values lying between them. That is why, the Voigt–Reuss–Hill bulk modulus, *B*_VRH_^*X*^, is defined as
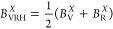
20

Similarly, shear modulus can be defined as
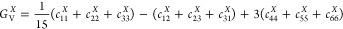
21or
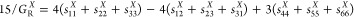
22

Again, *G*_R_^*X*^ < *G*_V_^*X*^ and real values should lie in between. Thus, the Voigt–Reuss–Hill shear modulus is defined as
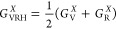
23

Taking into account the small difference between isentropic and isothermal elastic constants and in order to simplify the analysis of the results, *B*^T^ and *G*^T^ will be used to compute the other properties presented in this work.

**Figure 4:**
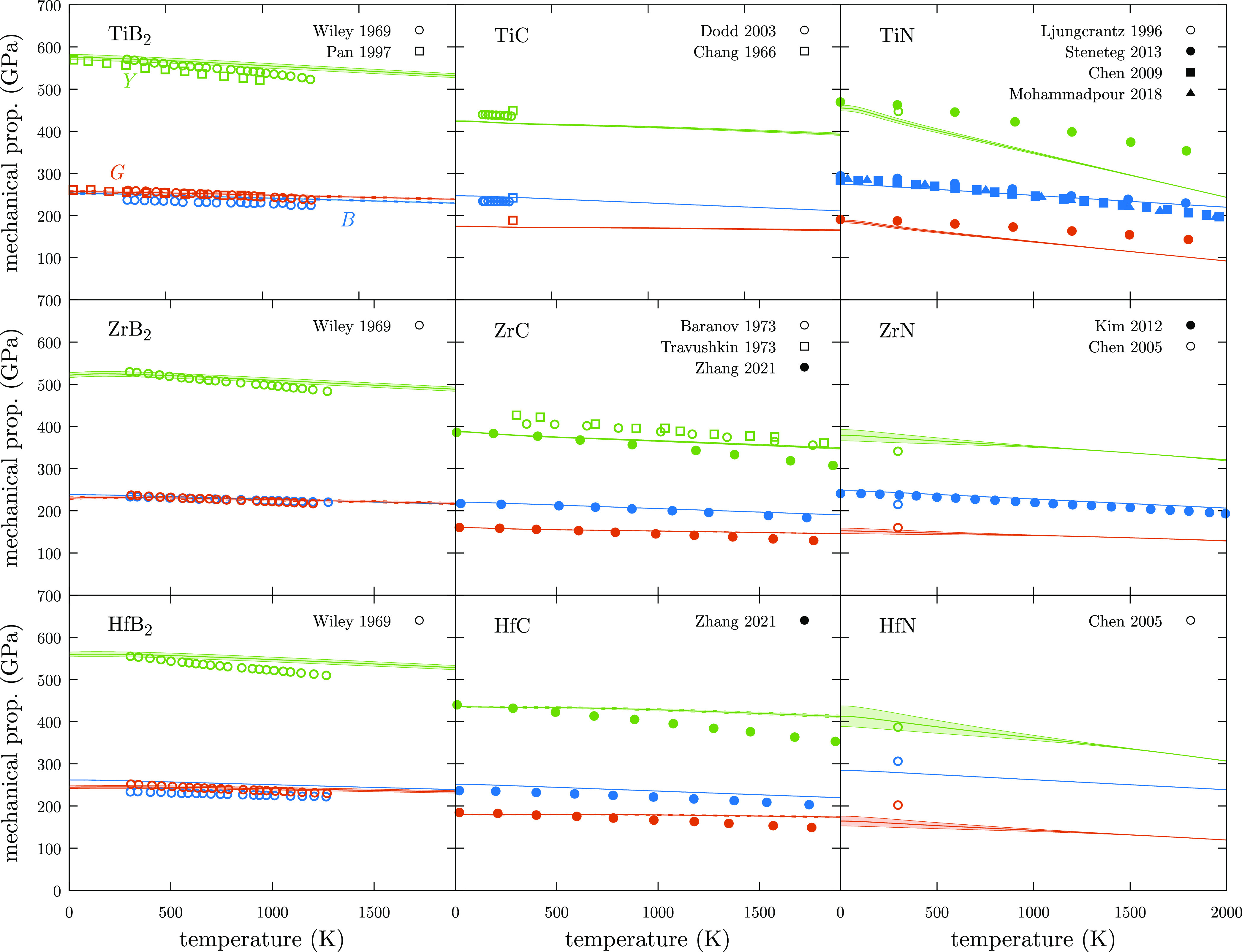
Bulk modulus, *B* (blue), shear modulus, *G* (orange), and Young’s modulus, *Y* (green), for UHTCs. Voigt and Reuss values are depicted with dashed lines, and Voigt–Reuss–Hill values are depicted with a solid line. The area ranged between Voigt and Reuss values has also been filled with the same color than the property. Open points represent experimental measurements while filled points represent calculated values.

The comparison of simulations with experimental values for *B* and *G* is not a simple task (see Figure 4). Most of the time, experimental measurements are obtained from polycrystalline samples in which porosity plays an important role, modifying their mechanical properties. There are different models that correlate the mechanical properties of the fully dense material and the porosity with the mechanical properties of actual samples.^41^ Here, the Gibson and Ashby equation^41^ was adopted to compare the experimental *B*, *G*, and Young’s modulus (*vide infra*), *Y*, with the theoretical results, if the porosity of the sample was reported. For instance, predicted values are in excellent agreement with experimental measurements^42,43^ for the *B* and *G* of borides, once porosity is considered. A similar trend is also found for TiC where experimental values are also available.^35,44^ Larger deviations with respect to experimental values are found for HfN where *G* is underestimated around 15% at 298 K. Comparing with other previous theoretical results also helps to demonstrate how this new approach can be not only accurate but also how it can substantially reduce the computational effort. For instance, the values obtained for *B* and *G* match well with the results reported by methods based on the QHA in which phonon calculations are performed for all distorted structures for ZrC and HfC,^39^ TiN,^45,46^ and ZrN.^47^

#### 3.4.2 Young’s Modulus and Poisson Ratio

Young’s modulus, *Y*, is a directional property; however, it can be initially assumed isotropic in order to extract a single approximate value for each compound
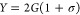
24where σ is the isotropic Poisson ratio
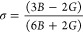
25

Similarly to *B* and *G*, our calculations are in good agreement with experimental values, when available for *Y* (Figure 4). Calculated values for borides^42,43^ present a maximum relative error around 7% at high temperatures, which is very small considering: (i) the values that are obtained using a very simple model to take into account the porosity of the sample^48^ and (ii) high-order force constants, which are not calculated here, can play an important role at high temperatures. Similar trends are observed for TiC^35,44^ and ZrC,^49,50^ where predicted *Y* values are slightly underestimated, but follow the same trend as experimental measurements. If experimental reports were not available, previous theoretical works were used to evaluate the results obtained with this new high-throughput approach. No significant discrepancies were found when *Y* values were compared with the results obtained with methods based on the QHA (see ZrC and HfC^39^). Larger differences were found for TiN where Steneteg *et al.* used molecular dynamics to study the mechanical properties of TiN.^38^ Nevertheless, *Y* experimental values for TiN single crystals at room temperature are between 445 and 449 GPa,^51^ which are close to the values calculated in this work.

To the best of our knowledge, there are not many experimental studies of the temperature dependence of the Poisson ratio. For instance, Wiley *et al.* explored the Poisson ratio of the borides of the group IV up to 1300 K.^42^ Our results are not only in agreement at room temperature but also reproduce the very small variation that this property presents in a large temperature range (Table 1). When compared with borides, the temperature dependence is slightly higher for carbides but is even larger in TiN and HfN. This trend is also observed in the wider range of experimental values previously reported (Table 1).

**Table 1:** Comparison of the Calculated Poisson Ratio for UHTCs in This Work in a 0–2000 K Temperature Range and Experimental Reported Values (Exp.)

	B		C		N	
	this work	exp.^42^	this work	exp.^52,53^	this work	exp.^52,53^
Ti	0.11–0.12	0.10–0.11	0.18–0.21	0.17–0.19; 0.19	0.22–0.36	0.30; 0.22
Zr	0.12–0.13	0.11–0.12	0.18–0.21	0.19–0.26; 0.20	0.23–0.25	0.19–0.25; 0.26
Hf	0.13–0.14	0.12–0.13	0.18–0.21	0.16–0.18; 0.16	0.24–0.32	0.26–0.35; 0.17

#### 3.4.3 Hardness

**Figure 5:**
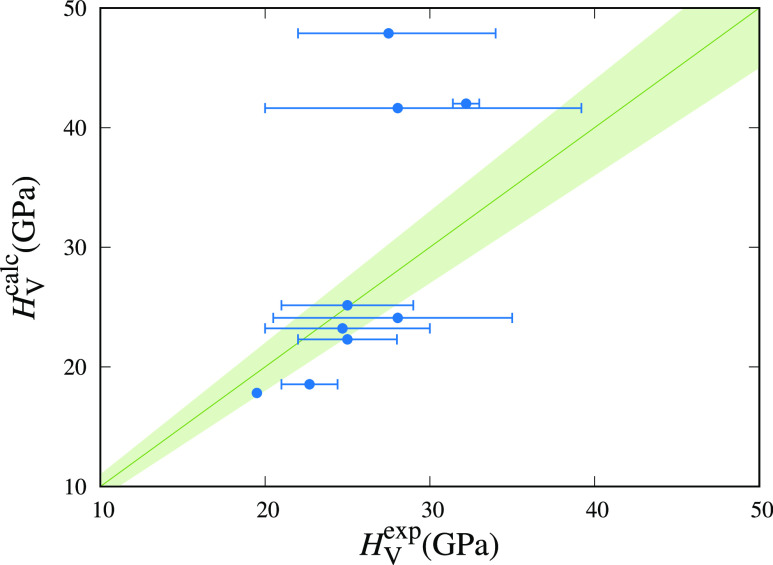
Comparison between calculated, *H*_V_^calc^, and experimental, *H*_V_^exp^, hardness. Green area represents the ±10% relative error with respect to experimental values. Blue error bars represent the dispersion of reported values for *H*_V_^exp^.

Hardness is probably one of the most difficult mechanical properties to predict and compare with experimental data. It presents not only a dependency with the porosity or grain size but also with other variables related to the measurement, such as indentation type (nano or micro), load, and time. During the last two decades, different models have been proposed to predict the hardness of materials. Most of them assume isotropic conditions which could overestimate the hardness of some materials, depending on the crystal plane exposed on the surface.^54^ Moreover, porosity and indentation load tend to reduce the values obtained for hardness. Here, hardness is calculated using the approach proposed by Tian *et al.*(55)
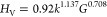
26where *k* is the Pugh’s modulus, which is defined as the ratio between the shear, *G*, and the bulk modulus, *B*. In eq 26, the constants are adjusted to obtain *H*_V_ in GPa units. At room temperature, borides seem to be the most overestimated values obtaining 47, 41, and 42 GPa for TiB_2_, ZrB_2_, and HfB_2_ when experimental values range between 34–22,^48^ 39–20,^56,57^ and 33–31.4 GPa,^58^ respectively (Figure 5). TiC stands as a good example of the different values that can be obtained for hardness, depending on the plane and orientation of the crystal.^53,59^ For instance, the TiC(100) plane on the [110] direction presents a micro-Vickers hardness of 34.9 GPa while the values for the (110) plane at the [100] direction is 23.24 GPa.^59^ Using the approach proposed by Tian, the calculated value (24.1 GPa) is in the range experimentally reported at room temperature. Similarly, calculated hardness for ZrC (23.2 GPa) and HfC (25.2 GPa) is in good agreement with the experimental measurements.^60−62^ To the best of our knowledge, there are not many experimental works which study the hardness of nitrides; however, we have found that calculated values are slightly lower than the homologue carbides and are close to the reported values for TiN,^63^ ZrN,^64^ and HfN.^36^

If predicting hardness is a difficult task because of the wide range of experimental variables, capturing the temperature dependence of this property is even more of a challenge. Hardness changes with temperature. Especially at high temperatures, hardness is controlled by creep due to dislocation diffusion phenomena. The activation energy for creep can be calculated from
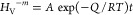
27where *Q*, *R*, *T*, and *t* are the activation energy for creep, the gas constant, the temperature, and the loading time, respectively, and *m* and *A* are constants.^65^ When Arrhenius plots are used to study hardness in a temperature range, different regions are identified for many UHTCs.^59,60^ These regions are linked to a brittle–ductile transition and the different mechanisms that govern the deformation during the indentation at different temperatures.^60^ Activation energies for creep for borides and carbides have been reported to be around 80 kcal/mol and most of the experimental studies obtain values for *m* between 4 and 5.^59,60^ These values can be combined with our predictions at room temperature to calculate the pre-exponential constant and obtain good trends for hardness below 1000 K.

### 3.5 Anisotropy

Dislocation dynamics or phase transformations are some examples in which anisotropy plays a relevant role. Quantification of crystal anisotropy has been a subject of debate since Zener introduced the first anisotropy index.^66^ Here, the universal elastic anisotropy index^67^
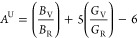
28and the log-Euclidean anisotropy index^68^
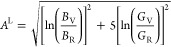
29are used to calculate the temperature-dependent anisotropy of UHTCs (Table 2).

**Table 2:** Anisotropy Indexes *A*^U^ and *A*^L^ for UHTCs in the 0–2000 K Temperature Range

	B		C		N	
	*A*^U^	*A*^L^	*A*^U^	*A*^L^	*A*^U^	*A*^L^
Ti	0.13–0.11	0.05–0.04	0.03–0.09	0.01–0.04	0.19–0.00	0.08–0.00
Zr	0.15–0.14	0.06	0.03–0.07	0.01–0.03	0.44–0.07	0.19–0.03
Hf	0.14–0.13	0.06	0.05–0.11	0.02–0.05	0.76–0.02	0.32–0.01

Borides, carbides, and nitrides, in general, present relatively low anisotropy indexes if they are compared with other materials.^68^ While the reported *A*^U^ and *A*^L^ for some cubic systems can be as low as 10^–3^, some triclinic and monoclinic materials present *A*^U^ values higher than 10^2^.^68^ Both indexes point to the nitrides as the most anisotropic materials, followed by the borides and finally the carbides (Table 2). These indexes are usually obtained from 0 K calculated mechanical properties; however, their behavior at high temperatures can be calculated using our approach. For instance, anisotropic indexes slightly increase with temperature for carbides, while they are reduced for borides and nitrides. Boride experiments change very little in their anisotropic indexes in the 0–2000 K range; however, both *A*^U^ and *A*^L^ are drastically reduced for nitrides.

**Figure 6:**
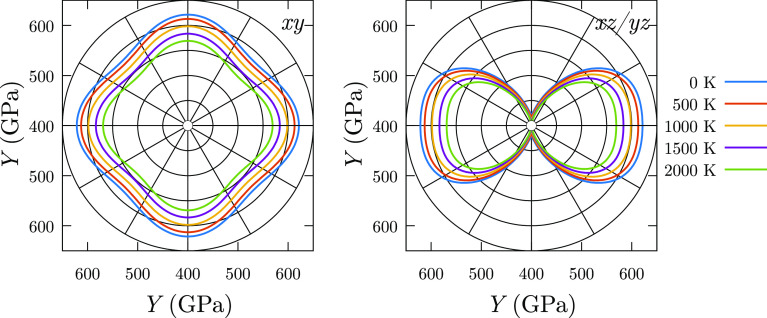
Anisotropic behavior of *Y* for TiB_2_ at different temperatures. Left and right panels correspond to *xy*, *xz*, and *yz* planes, respectively.

Anisotropy indexes give relevant information about the general behavior of the material; however, some properties such as *Y*, *G*, and σ can be directly calculated as a function of the crystallographic direction. This information is extremely valuable in order to predict the behavior of single-crystal materials in a specific direction or the limits of these properties in polycrystalline samples. There are already different packages that calculate and represent the anisotropic nature of some mechanical properties.^69^ In this work, ELATE package^70^ has been combined with our framework to explore the temperature-dependent anisotropic nature of some mechanical properties. As an example, the directional and temperature dependence of *Y* is plotted for TiB_2_ in Figure 6, where *Y* is a 33% lower in *c* with respect to *a* and *b*.

**Figure 7:**
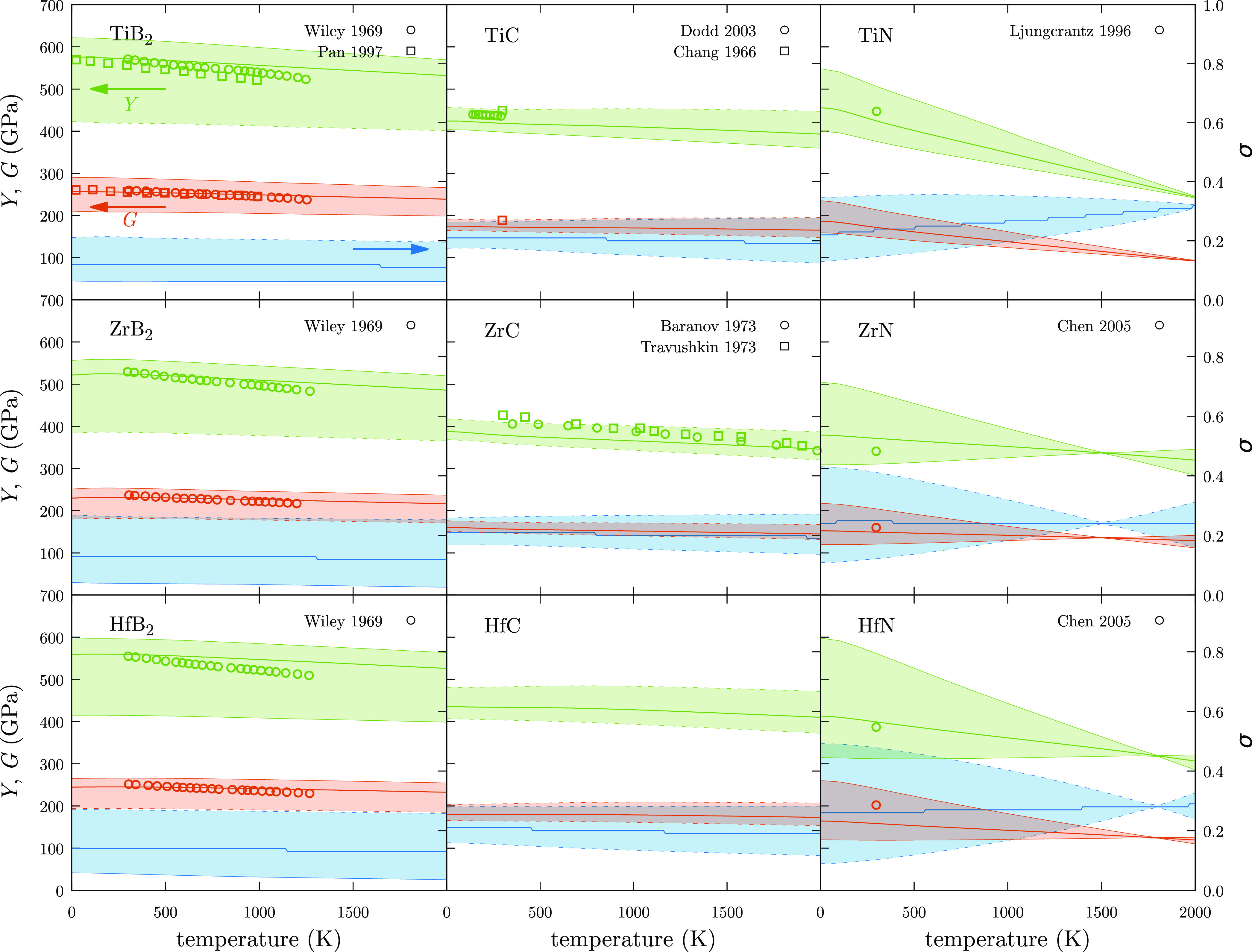
Anisotropic Poisson ratio, σ (blue), shear modulus, *G* (orange), and Young’s modulus, *Y* (green), for UHTCs. Solid lines represent isotropic values calculated in the previous section. Dashed lines represent the upper and lower limit for each property. The area ranged between upper and lower limits has also been filled with the same color than the property. Open points represent experimental measurements.

The same approach has been followed for the rest of UHTC materials studied in this work, not only for *Y* but also for *G* and σ (Figure 7). The colored area for each property at each temperature is delimited by the maximum and minimum values (dashed lines) predicted for *Y* (green), *G* (orange), and σ (blue). Results in Figure 7 are in good agreement with the trends extracted from anisotropy indexes. Carbides are the group of materials with a lower variability in their properties, which correspond to lower anisotropy indexes than borides and nitrides. Moreover, the difference between the maximum and minimum values in carbides slightly increases with temperature, following the same trend as the anisotropic indexes. Similarly to values in Table 2, the amplitude between the maximum and minimum for each property in borides remains almost constant with temperature, while a fast reduction of the amplitude can be observed for nitrides. Some singular points can be observed for nitrides, where the maximum and minimum values are the same at a given temperature. These points represent an inversion in the direction where the maximum and minimum values of a specific property can be observed. For instance, *Y* maximum values are observed in the [100] direction up to 1500 K approximately. For temperatures higher than 1500 K, this trend is different and the minimum value for *Y* is obtained in the [100] direction. The same phenomenon is observed in TiN and HfN around 2000 and 1800 K, respectively. Figure 7 shows a good approach to visualize the potential scattering in experimental measurements depending on the crystallinity, direction, and temperature in which the property has been measured. Experimental data already plotted in Figure 4 are always in between the maximum and minimum limits established for each property at each temperature. Only one experimental point is slightly out of the delimited area for ZrC. This very small deviation could be due to the well-known underestimation of bond strength by GGA functionals.

### 3.6 Thermodynamic and Thermal Properties

#### 3.6.1 Heat Capacity

Thermodynamic properties such as specific heat at constant volume, *C*_*V*_, are calculated including phonon, *C*_*V*_^ph^, and electronic, *C*_*V*_^el^, contributions
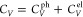
30

Phonon contribution is calculated as
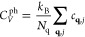
31where
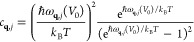
32

**Figure 8:**
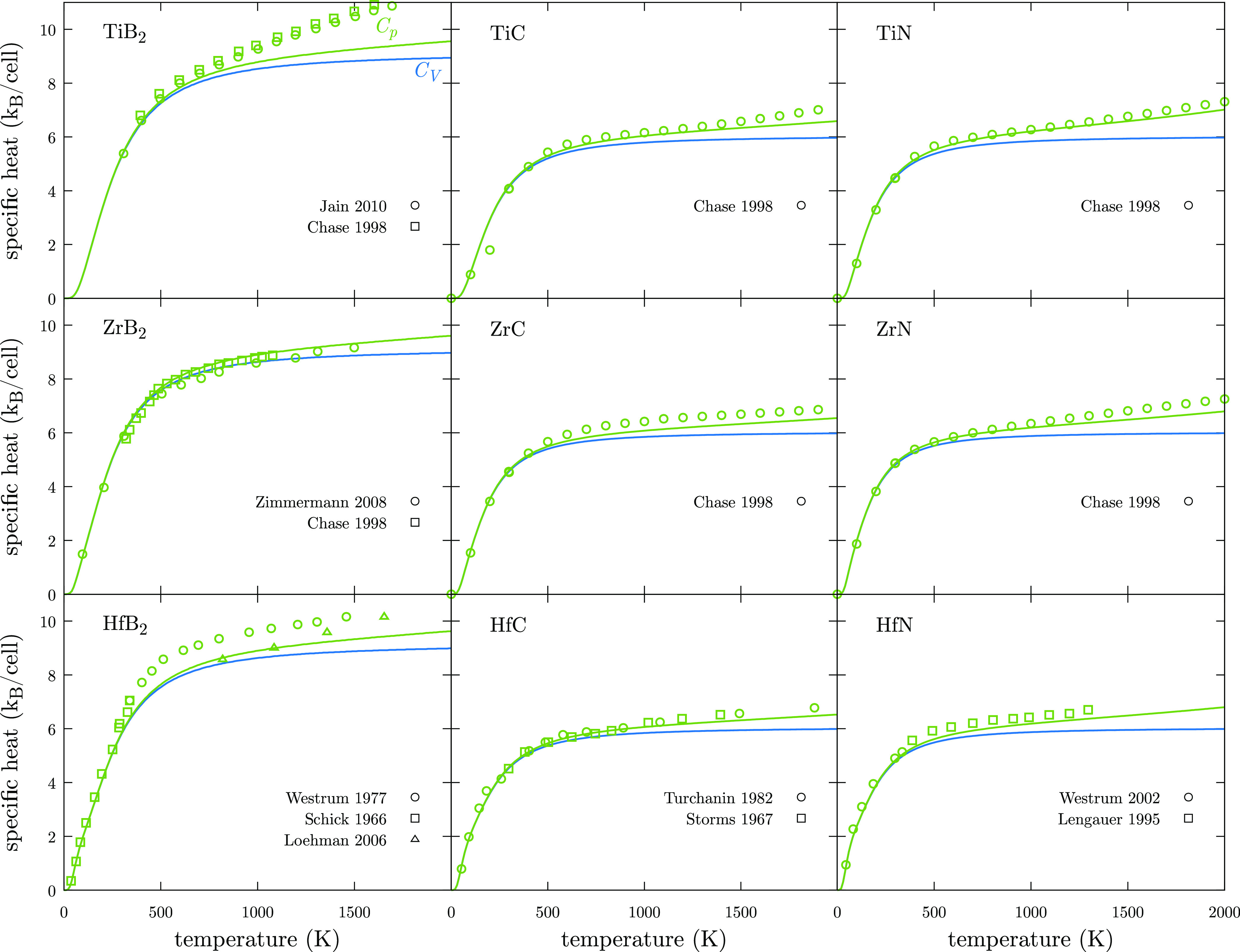
Heat capacity at constant volume, *C*_*V*_ (blue), and at constant pressure, *C*_*p*_ (green) for UHTCs. Solid lines represent calculated values while open points represent experimental measurements.

Following the free electron gas approximation, the electronic contribution to heat capacity is a linear function with respect to the temperature
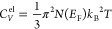
33where *N*(*E*_F_) is the DOS at the Fermi level. Moreover, the specific heat at constant pressure, *C*_*p*_, which is more experimentally accessible, is calculated as
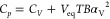
34where *V*_eq_ is the equilibrium volume for a given temperature and α_V_ is the volumetric lattice thermal expansion. In most cases, specific heats match experimental values well^71−80^ (Figure 8). Only small deviations, below 10% error, are found for TiB_2_ and HfB_2_. It has been pointed out that the origin of this difference is the importance of higher-order lattice anharmonic vibrations at higher temperatures, which are not included under the frame of the QHA.^34^ Anharmonicity can play an important role at very high temperatures through different phenomena such as phonon–phonon or electron–phonon scattering. Including the effect of phonon lifetimes represents the most important limitation of this methodology and stands as the next challenge in order to obtain a more accurate picture of the mechanical response of materials at temperatures close to their melting point. For instance, Mellan *et al.* have studied the effects of phonon–phonon and electron–phonon scattering in the calculation of the thermal conductivity of ZrC.^81^ However, due to the extremely expensive computational costs, very few studies include anharmonicity effects in the prediction of mechanical properties.^82^

#### 3.6.2 Grüneisen Parameter

Grüneisen parameter, γ, is a good measurement of the compressibility of the phonons and it is often used to estimate the anharmonicity of the vibrations in the crystal (Figure S4). As other properties already discussed, γ is a tensorial magnitude which depends on the direction of the tension–compression. Here, the average Grüneisen parameter was calculated based on an isotropic expansion–compression of the solid
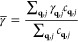
35where
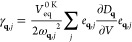
36*D*_**q**_ is the dynamical matrix for a wave vector, **q**, ω_**q**,*j*_ is the vibrational frequency, and *e*_**q**,*j*_ is the eigenvector for phonon branch, *j*. The detailed procedure can be found in refs (10) and (23). It seems that the calculated values for borides follow the same trend as experimental results reported by Wiley *et al.*,^42^ with γ̅ being constant over 500 K. Quantitatively, calculated values seem to slightly overestimate Wiley *et al.* results. However, values reported by Ajami and MacCrone^83^ and Dodd *et al.*(44) at 300 K are in good agreement with the predictions, indicating that the error of the calculation is lower than the deviation of the experimental measurements. In addition to the comparison with the experimental results, different conclusions can be extracted from Figure S4. No large changes are observed with the temperature for borides, carbides, or nitrides. For instance, γ̅ for TiN seems to the one with the strongest dependence with respect to the temperature. If families are compared to each other, nitrides present the higher values for γ̅ and then carbides and finally borides.

#### 3.6.3 Thermal Expansion

Some of the previous thermodynamic properties can be accurately obtained with low computationally demanding methods such as Gibbs.^8^ However, the accurate prediction of thermal expansion coefficient requires the calculation of the free-energy surface which is computationally demanding. Alternatives to the standard QHA^9,10^ such as QHA3P^23^ can reduce the computational cost, but QHA3P has been only used for calculating volumetric thermal expansion and cannot capture the anisotropic nature of the material, when isotropic deformations are applied. The framework developed in this work fills this gap, capturing the anisotropy of the system through the calculation of linear thermal expansion coefficients and reducing the computational cost to the same level than QHA3P.

Linear and volumetric thermal expansion coefficients, α_*i*_, are calculated using the free-energy curves obtained for the temperature-dependent elastic constants
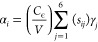
37where *C*_ϵ_ is the heat capacity at constant strain ϵ, γ_*j*_ is the Grüneisen parameter along different *j* directions, and *s*_*ij*_ are the elastic compliance constants.

**Figure 9:**
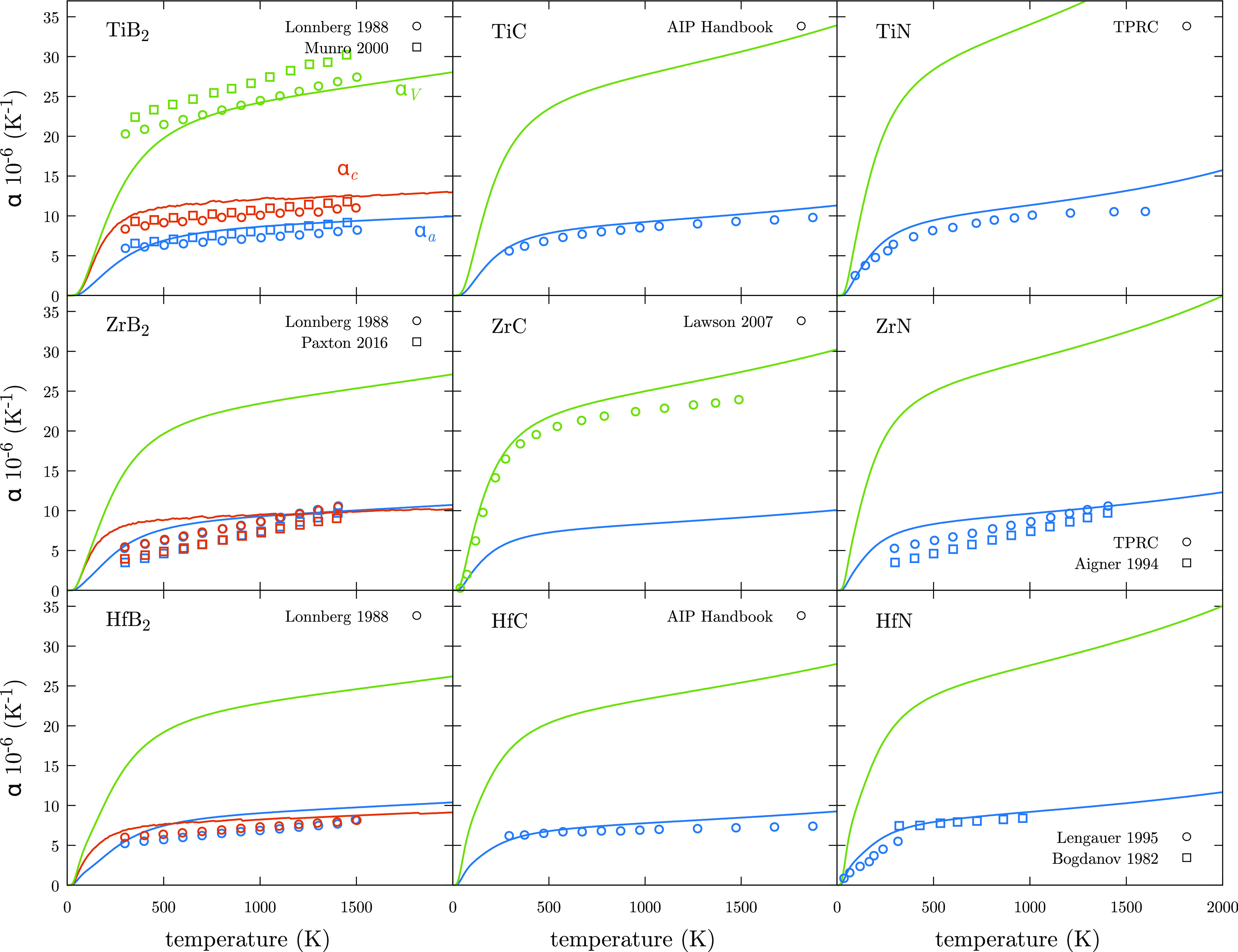
Volumetric thermal expansion coefficient, α_V_ (green), and linear thermal expansion coefficients, α_*a*_ (blue) and α_*c*_ (orange), for UHTCs. Solid lines represent calculated values while open points represent experimental measurements.

When calculated values are compared with experiments (Figure 9), good agreement was obtained for most carbides^84,85^ and nitrides,^86−89^ while larger deviations were found for some of the borides.^48,89,90^ As it is defined in eq 37, α depends on *C*_ϵ_, *s*_*ij*_, and γ. It has been proven in the previous sections that accurate values were obtained for *C*_ϵ_ and *s*_*ij*_ (*c*_*ij*_). However, higher deviations were found for γ. Thus, the main source of error stems from the description of the anharmonicity of the material which is not completely well-described by the QHA.

## 4 Conclusions

In this work, mechanical, thermal, and thermodynamic properties of UHTCs at high temperatures have been exhaustively explored using a new theoretical framework. The main advantage of this new approach relies on the drastic reduction of the computational cost without losing accuracy. This strategy gives a computational inexpensive solution to the difficulty of experimentally obtaining their mechanical properties at temperatures close to their melting point, which was hampering the fast technological development of this family of materials. Very good agreement between experiments and calculations was found not only for the elastic constants but also for other mechanical properties such as *B*, *G*, *Y*, or σ, when data are available. Although hardness is a property that also depends on plastic deformation, good trends were also predicted. While approximations or frameworks with similar computational cost only predict isotropic or averaged properties, this new approach also predicts anisotropic properties describing directional mechanical properties such as *Y* or establishing upper and lower limits for polycrystalline materials, which are extremely valuable data in industry to determine their applicability. Thermodynamical and thermal properties were also explored, obtaining, in most cases, good agreement with available experimental data. To the best of our knowledge, this study represents one of the most comprehensive characterizations of this family of compounds with direct implications in different technologies. In addition to quantity and quality of data for the characterization of UHTCs, this new theoretical framework facilitates the access to temperature-dependent mechanical properties, extremely expensive to obtain with current computational approaches. This new framework opens the door to characterize, design, or discover new materials whose application or importance rely on their mechanical properties at different temperatures, from coating or material behavior during their processing to mineral’s response to seismic waves.

## Supporting Information Available

Phonon dispersion curves; comparison of HfC elastic constants; Born stability criteria; and Grüneisen parameters (PDF)
