# Supplementary material for: High-Throughput Screening of the Thermoelastic Properties of Ultrahigh-Temperature Ceramics
Source: ACS Appl Mater Interfaces. 2021 Jun 16;13(25):29843–57. doi: 10.1021/acsami.1c08832 (PMC8509953; doi:10.1021/acsami.1c08832)
Supplement: Supplementary file 1 — am1c08832_si_001.pdf [file am1c08832_si_001.pdf]

Supporting Information: High-throughput screening of the thermoelastic properties of ultra-high temperature ceramics

Pinku Nath,[†] Jose J. Plata,[*,‡] Julia Santana-Andreo,[‡] Ernesto J. Blancas,[‡] Antonio M. Márquez,[‡] and Javier Fernández Sanz[‡]

†*School of Chemical Engineering and Physical Science, Lovely Professional University, India*

‡*Departamento de Química Física, Facultad de Química, Universidad de Sevilla, Seville, Spain*

E-mail: jplata@us.es

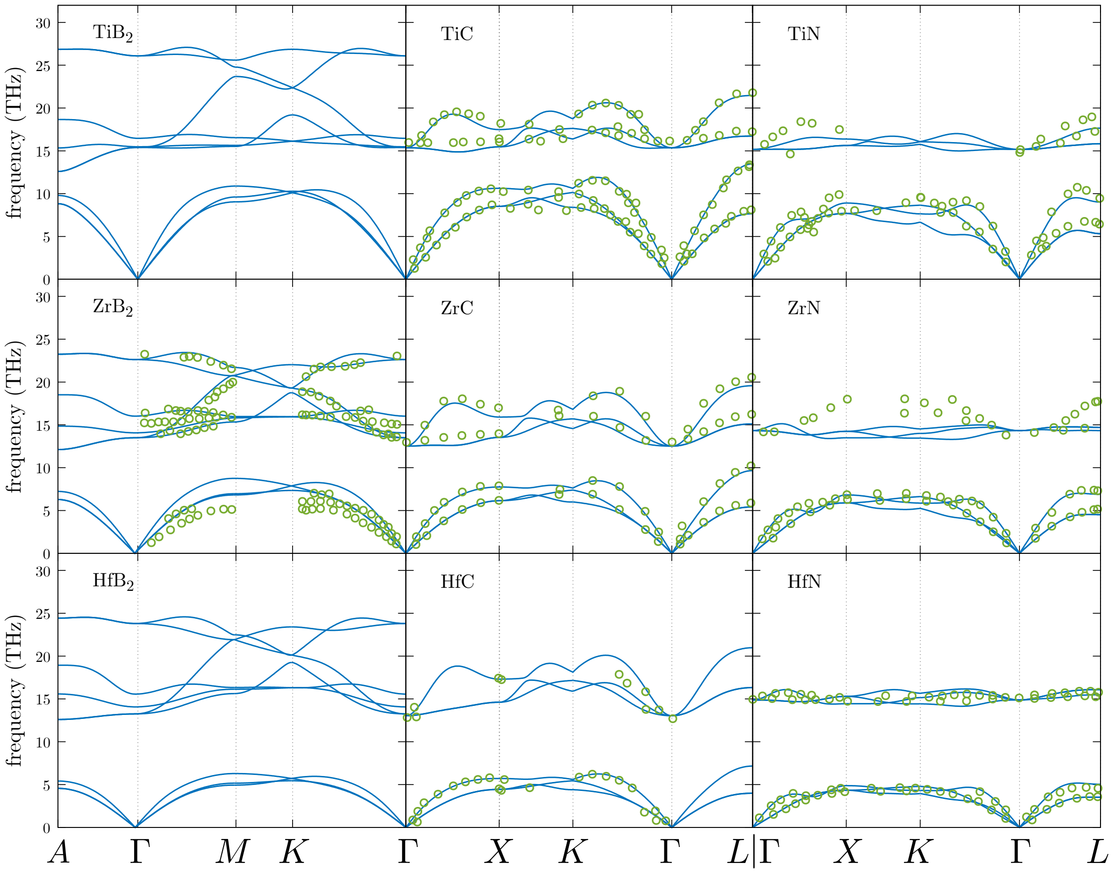

Figure S1: Phonon dispersion curves for ultra high temperature borides, carbides and nitrides along high symmetry directions in the Brillouin zone. Experimental points for ZrB_2,[S1] NbB_2,[S1] TiC,[S2] TiN,[S3] ZrC,[S4] ZrN,[S5] HfC,[S6] HfN[S7] are shown with green circles.

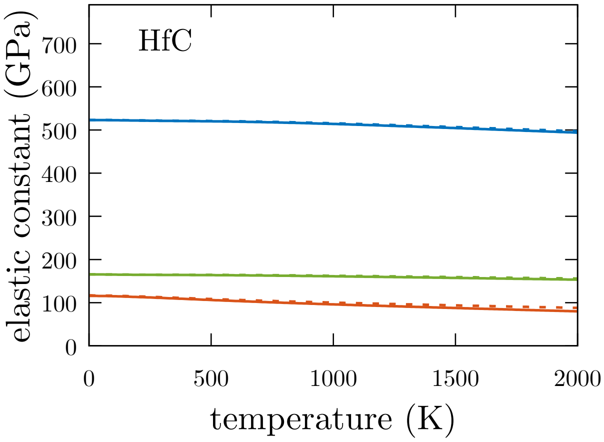

Figure S2: Comparison of the elastic constants obtained using the approach developed in this work (solid line) and calculating the phonon spectra of each distorted cell (dashed lines) for HfC. Colors: c_{11} = blue; c_{12} = orange; c_{13} = yellow; c_{33} = purple; c_{44} = green.

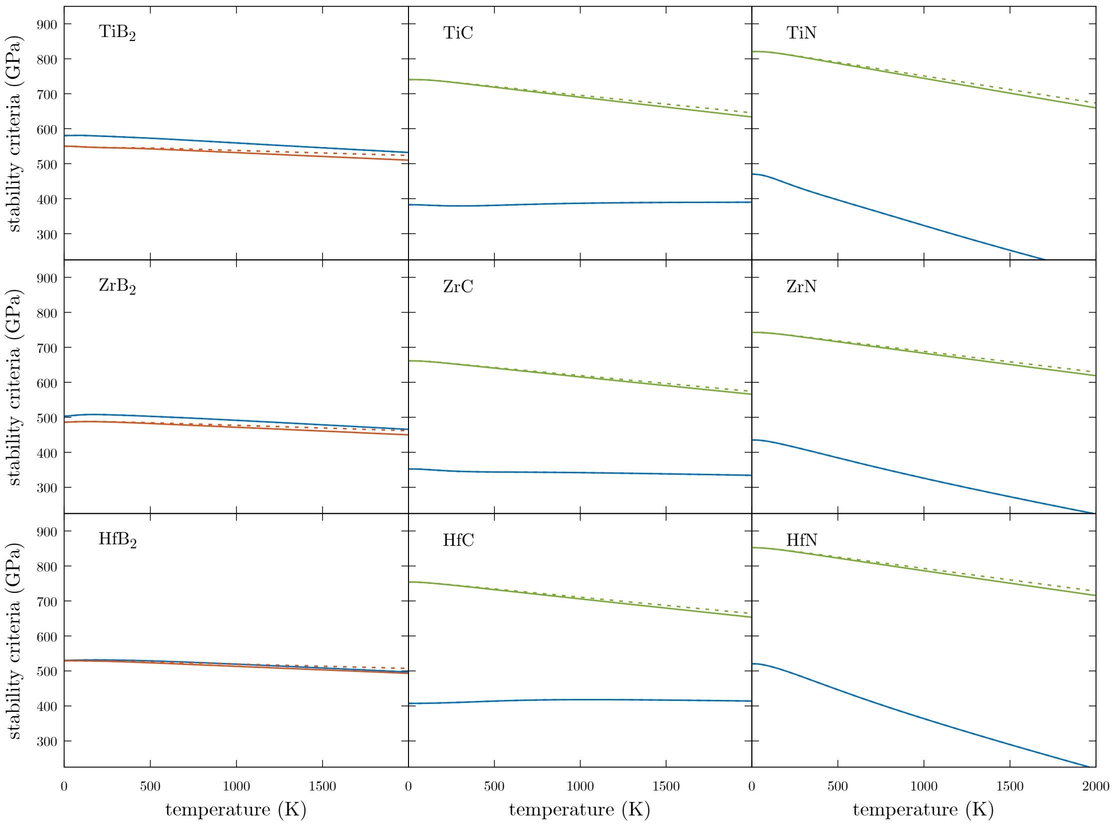

Figure S3: Born stability criteria using isothermal (solid lines) and isentropic (dashed lines) elastic constants for UHTCs. Colors: $c_{11} - c_{12}$ = blue; $\left(\left(c_{11} + c_{12}\right) c_{33} - 2c_{13}^2\right)^{1/2}$ = orange; $c_{11} + 2c_{12}$ = green.

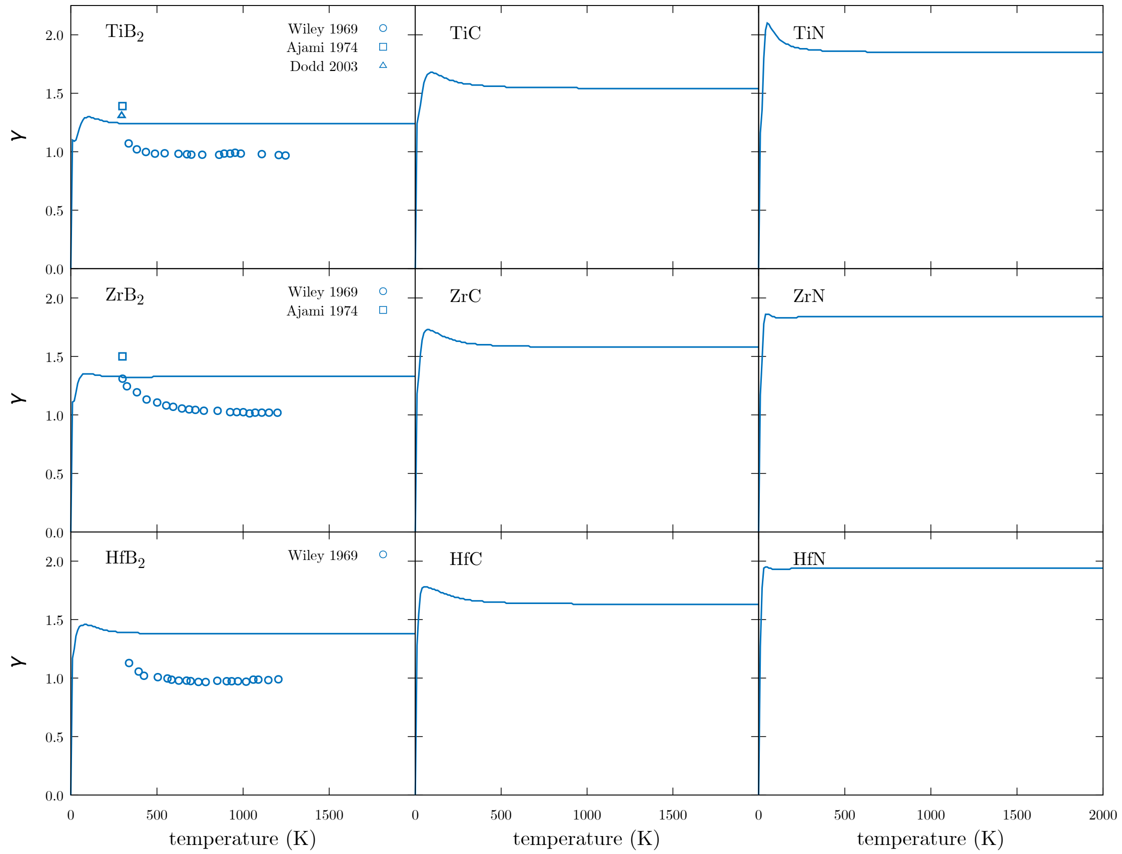

Figure S4: Calculated averaged Grüneisen parameter, $\bar{\gamma}$ (solid lines), for UHTCs. Experimental data for borides[S8,S9] are shown with open points.

References

(S1) Aizawa, T.; Hayami, W.; Otani, S. Surface phonon dispersion of ZrB_2(0001) and NbB_2(0001). *Phys. Rev. B* **2001**, *65*, 024303.

(S2) Pintschovius, L.; Reichardt, W.; Scheerer, B. Lattice dynamics of TiC. *J. Phys. C: Solid State Phys.* **1978**, *11*, 1557–1562.

(S3) Kress, W.; Roedhammer, P.; Bilz, H.; Teuchert, W. D.; Christensen, A. N. Phonon anomalies in transition-metal nitrides: TiN. *Phys. Rev. B* **1978**, *17*, 111–113.

(S4) Weber, W. Lattice Dynamics of Transition-Metal Carbides. *Phys. Rev. B* **1973**, *8*, 5082–5092.

(S5) Christensen, A. N.; Dietrich, O. W.; Kress, W.; Teuchert, W. D. Phonon anomalies in transition-metal nitrides: ZrN. *Phys. Rev. B* **1979**, *19*, 5699–5703.

(S6) Smith, H. G.; Glazer, W. Proceedings of the International Conference on Phonons. Rennes, Frances, 1971.

(S7) Christensen, A. N.; Kress, W.; Miura, M.; Lehner, N. Phonon anomalies in transition-metal nitrides: HfN. *Phys. Rev. B* **1983**, *28*, 977–981.

(S8) Wiley, D. E.; Manning, W. R.; Hunter, O. Elastic properties of polycrystalline TiB_2, $ZrBi_2$ and HfB_2 from room temperature to 1300 K. *J. Less-Common Met.* **1969**, *18*, 149 – 157.

(S9) Ajami, F. I.; MacCrone, R. K. Thermal expansion, Debye temperature and Gruneisen constant of carbides and nitrides. *J. Less-Common Met.* **1974**, *38*, 101 – 110.
